# Customizable Implant-specific and Tissue-Specific Extracellular Matrix Protein Coatings Fabricated Using Atmospheric Plasma

**DOI:** 10.3389/fbioe.2019.00247

**Published:** 2019-09-27

**Authors:** Fei Tan, Mohamed Al-Rubeai

**Affiliations:** ^1^Department of Otolaryngology - Head & Neck Surgery, Shanghai East Hospital, Tongji University School of Medicine, Shanghai, China; ^2^School of Chemical and Bioprocess Engineering, and Conway Institute of Biomolecular and Biomedical Research, University College Dublin—National University of Ireland, Dublin, Ireland; ^3^The Royal College of Surgeons of England, London, United Kingdom; ^4^Department of Biology, Cihan University-Erbil, Erbil, Iraq

**Keywords:** atmospheric plasma, implant, coating, collagen, laminin, bone, nerve

## Abstract

Progression in implant science has benefited from ample amount of technological contributions from various disciplines, including surface biotechnology. In this work, we successfully used atmospheric plasma to enhance the biological functions of surgical implants by coating them with extracellular matrix proteins. The developed collagen and laminin coatings demonstrate advantageous material properties. Chemical analysis by XPS and morphological investigation by SEM both suggested a robust coating. Contact angle goniometry and dissolution study in simulated body fluid (SBF) elicited increased hydrophilicity and physiological durability. Furthermore, these coatings exhibited improved biological interactions with human mesenchymal and neural stem cells (NSCs). Cell adhesion, proliferation, and differentiation proved markedly refined as shown by enzymatic detachment, flow cytometry, and ELISA data, respectively. Most importantly, using the pathway-specific PCR array, our study discovered dozens of deregulated genes during osteogenesis and neurogenesis on our newly fabricated ECM coatings. The coating-induced change in molecular profile serves as a promising clue for designing future implant-based therapy. Collectively, we present atmospheric plasma as a versatile tool for enhancing surgical implants, through customizable implant-specific and tissue-specific coatings.

## Introduction

Surgical implants are the quintessence of modern medicine. They represent a unique therapeutic modality owing to their interdisciplinarity. They are designed to replace missing body parts, to support damaged organs and tissues, or to enhance deficient biological functions (van Eck et al., 2009). Depending on the type of target tissue, implants can be roughly categorized into those repairing hard tissue and those restoring soft tissue. In head and neck surgery, the two best examples are bone anchored hearing aid (BAHA) and cochlear implant (CI), which influence bone and nerve tissue, respectively (Gaylor et al., 2013; Ghossaini and Roehm, 2019). Metals and polymers are the dominant classes of biomaterial used in these implants. Metals possess exceptional mechanical properties and corrosion resistance (Spriano et al., 2018), whereas polymers feature extraordinary flexibility and long-term stability (Teo et al., 2016). However, none of these materials is functionally perfect, as each has its advantageous and disadvantageous properties. Thus, there is constant need and drive to enhance the current surgical implants.

The surface of a surgical implant is the key area where implant-tissue reaction occurs. Biotechnology targeting the implant surface can promote the performance profiles of surgical implants. Recent innovations in surface biotechnology have demonstrated at least four intriguing strategies: substitute biomaterial, surface modification, drug delivery, and coating (Tan et al., 2013). Coating normally uses an entirely different material from the underlying surgical implant, attempting to combine the advantages of both layers. For example, from an osteogenic perspective, coating the intracortical screw of BAHA with ceramic can significantly improve its osteoconductivity while maintaining the implant's high mechanical strength (Sanden et al., 2002; Tan et al., 2012a). From a neurogenic perspective, coating the electrode of CI with conducting polymer can greatly enhance its neural biocompatibility without affecting the implant's electrical conductivity (Quigley et al., 2009; Green et al., 2012). Therefore, a coating should be implant-specific to enhance its established therapeutic function.

Furthermore, an ideal coating should also be tissue-specific. The native extracellular matrix (ECM) happens to consist of a tissue-specific, highly complex network of proteins and polysaccharides which provide structural scaffolding and biochemical cues for surrounding cells, including stem cells (Theocharis et al., 2016). In addition, the main protein components of ECM: collagen, laminin, and fibronectin have substantial impact on tissue-specific stem cell morphogenesis, differentiation, and homeostasis (Frantz et al., 2010; Ahmed and Ffrench-Constant, 2016). For example, collagen promotes mesenchymal stem cell (MSC) proliferation and encourages osteogenic differentiation from MSCs (Somaiah et al., 2015). On the other hand, laminin enhances neural stem cell (NSC) migration, expansion, differentiation into neurons, and their derived neurite outgrowth (Flanagan et al., 2006). Many coating techniques have been explored to deposit proteins onto surgical implants. These include simple immersion (Rammelt et al., 2004), covalent immobilization (Ao et al., 2016), and chemical bonding (Hum and Boccaccini, 2018). These methods all share a common characteristic, i.e., non-thermality, due to the heat-sensitive nature of proteins.

Atmospheric plasma is an emerging non-thermal biotechnology. In simple term, non-thermal plasma is a technical, adjustable, and ambient version of thermal plasma (e.g., solar corona) (Tendero et al., 2006). Atmospheric plasma has been recently applied in medicine and the life sciences. Depending on the approach, application can involve direct, or indirect treatment (Fridman et al., 2008). Direct plasma treatments can assist in wound healing and skin rejuvenation in dermatology (Heinlin et al., 2011), and perform effective dissection and precise tissue removal in head and neck surgery (Metcalfe et al., 2017). Meanwhile, indirect plasma treatments exert the therapeutic effect mainly through processing the surface of biomedical devices (e.g., surgical implants). Recent examples cover most of the strategies in surface biotechnology: surface modification (Prasad et al., 2010; Tan et al., 2012b), drug delivery (Yoshida et al., 2013; Dowling et al., 2016), and implant coatings (Dowling et al., 2009; Tynan et al., 2009). We have successfully used atmospheric plasma as a surface modification method to activate ceramic implants, thereby achieving better osteogenesis (Tan et al., 2012b). However, coating surgical implants by atmospheric plasma remains an underexploited area, especially with ECM proteins.

The primary objective of this study was to establish implant-specific (metal and polymer) and tissue-specific (osteogenic and neurogenic) ECM coatings (collagen and laminin) facilitated by atmospheric plasma. The secondary objective was to compare coatings deposited using different techniques (atmospheric plasma vs. traditional adsorption). Particular emphasis was placed on material properties (physical and chemical), cellular interactions (MSC and NSC), and transcriptomic activities (osteogenesis and neurogenesis pathways). The final objective was to elucidate the molecular basis through which these novel coatings enhance tissue-specific differentiation from stem cells.

## Materials and Methods

### Atmospheric Plasma Set-Up

In order to simulate the metal and polymer surgical implants, grade V Titanium (Ti) alloy coupon (Lisnabrin Engineering Ltd., Cork, Ireland) and tissue culture grade polystyrene (PS) disk (Thermo Scientific, Surrey, UK) sized 20 × 20 × 1 mm were used, respectively. Surface roughness plays an important role in cell attachment and differentiation (Ponsonnet et al., 2003; Li et al., 2016). Since polystyrene surfaces tend to be relatively smooth, for comparison purposes, we created a rough surface via grit-polishing and grit-blasting Ti samples. The Ti alloy substrates were polished to a 1,200 grit size using silicon carbide paper. The polished samples were ultrasonically cleaned for 5 min to remove residual particulates, consecutively in acetone, methanol, and isopropyl alcohol (Sigma-Aldrich, Dorset, UK). They were then blasted by Al_2_O_3_ (Comco Incorporated, Burbank, USA) with a mean particle size of 100 μm using a microblasting platform (ENBIO, Dublin, Ireland) (Tan et al., 2012a). Finally, the roughened metal disks and as-received polymer specimens were immersed in deionized water (dI H_2_O) and ultrasonically cleaned for 5 min. They were dried at room temperature, stored in a desiccator, and prepared for the coating process.

**Figure 1:**
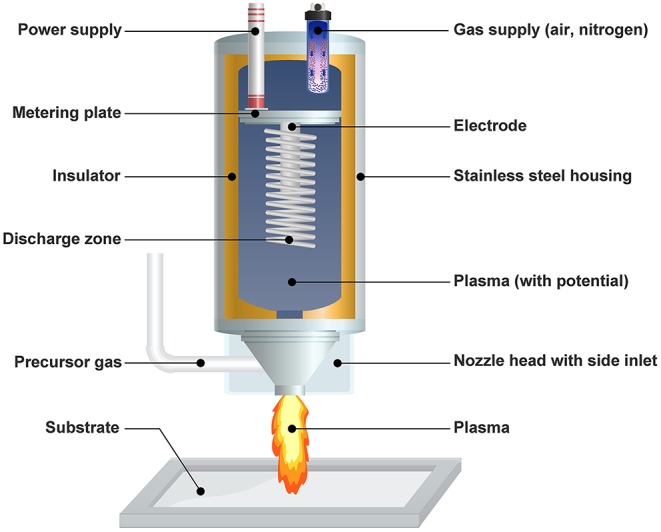
Cross-section diagram of the PlasmaPlus APPJ coating nozzle.

The PlasmaPlus® technology (Plasmatreat GmbH, Steinhagen, Germany) was used to facilitate coating formation (Bringmann et al., 2009; Dowling et al., 2009; Scopece et al., 2009). It is essentially an atmospheric pressure plasma jet (APPJ) based on air or nitrogen plasma (Figure 1). The Plasmatreater AS 400 laboratory system was modified for this study. The plasma was driven by a DC power supply operating at 23 kHz (280 V, 15 A). Clean dry industrial air with a flow rate of 35 l/min was used as ionization gas in the plasma torch. The plasma cycle time (PCT), an indicator of plasma intensity, was set at 100%. The APPJ was mounted on an X-Y-Z motion system, which traveled in a raster pattern at a line speed of 180 mm/s and a scanning interval of 1 mm. The plasma jet orifice was placed above the test substrates at a standoff distance of 15 mm.

### Deposition of ECM Coatings

**Figure 2:**
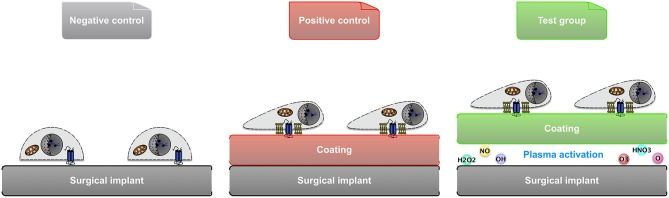
Illustration of the 3 groups of samples used in material comparison and cellular study: negative control (substrate only), positive control (coating by adsorption), and test group (coating facilitated by atmospheric plasma).

In order to obtain ECM coatings on the aforementioned implants, type I collagen from human fibroblasts, and laminin from human fibroblasts and epithelial cells co-culture were used, respectively (Sigma-Aldrich). A 10 μg/ml working solution of collagen or laminin was prepared. The former was diluted from stock using phosphate-buffered saline (PBS) and pH-adjusted to 7.2–7.6 using 0.1 M NaOH or 0.01 M HCl (Heino, 2007), whereas the latter was simply diluted using Hank's balanced salt solution (HBSS) (Kleinman, 2001). The above reagents all came from Fisher Scientific, Dublin, Ireland. All samples were divided into three groups: negative control (substrate only), positive control (coating by adsorption), and test group (coating by atmospheric plasma) (Figure 2).

In the test group, the Ti or PS substrates were each surface-activated by atmospheric plasma with ten passes. The collagen or laminin working solution was then sprayed immediately after plasma activation in order to maximize its efficacy. The ECM protein was applied in a nebulized form to the substrate using a piezoelectric sonication system (Breathnach et al., 2018). The nebulized collagen or laminin was sprayed for 3 s using nitrogen at a relatively low flow rate of 3 l/min. The amount of ECM protein solution delivered was ~3 ml per substrate.

In the positive control group, where the coating was created using an adsorption method, the stored metallic and polymeric specimens were placed in 6-well tissue culture plates (Sarstedt, Wexford, Ireland). Each well was immersed with 3 ml of collagen and laminin working solution, respectively. They were then incubated at 37°C for 20 min and rinsed 3 times with PBS or HBSS. Irrespective of the coating method, the coated samples were air dried under a laminar flow hood.

### Material Characterization of Coatings

The chemical composition of coatings was disclosed by X-ray photoelectron spectroscopy (XPS) analysis using VersaProbe XPS microprobe (ULVAC-PHI Inc., Kanagawa, Japan). This instrument provided dual beam charge neutralization. Measurement was conducted on three areas of each sample, and data were recorded as relative atomic percentage concentration.

The hydrophilicity of coatings was quantified using the static sessile drop technique at room temperature by a computer automated goniometer (OCA 20, Dataphysics, Filderstadt, Germany). In detail, the static contact angle of simulated body fluid (SBF) was measured for eight separate locations on each sample (Tan et al., 2012b). In order to obtain SBF with an ion concentration close to that of human blood plasma, reagent grade powders were dissolved in dI H_2_O at 37°C, and pH-adjusted to 7.4 using 50 mM Tris aminomethane and 45 mM HCl (Kokubo, 1991). All reagents were supplied by Fisher Scientific, Dublin, Ireland. Due to the limit of the equipment, surfaces with a contact angle <5° were all considered super-hydrophilic.

The topography of ECM protein coatings was documented using a white light optical profilometer (Wyko NT1100, Veeco, Cambridge, UK). The arithmetical mean roughness Ra was calculated using corresponding formula.

The collagen coatings acquired in this study were used as examples for morphological analysis, which included both scanning electron microscopy (SEM) and stereomicroscopy. On one hand, coating samples were coated with gold by a turbo pumped high-resolution sputter coater (K575X, EmiTech, Kent, UK) prior to examination under a scanning electron microscope (Quanta 3D FEG, FEI Ltd., Cambridge, UK) which operated at 5 kV and 6.66 pA with an observation angle at 90°. On the other hand, the robustness of collagen coatings was analyzed using the Sircol collagen assay (Biocolor, Carrickfergus, UK). This assay is based on the binding of a dye, Sirius Red, to the intact triple helix organization of native collagens. In brief, the Sircol dye reagent was mixed with dI H_2_O (1:1 v/v). The stained samples were then visualized under a SZX12 stereomicroscope (Olympus, Southend-on-Sea, UK).

The kinetic dissolution of collagen and laminin coatings was quantified using an enzyme-linked immunosorbent assay (ELISA). The coated implants were incubated at 37°C in SBF with a constant agitation at 6 rpm. At each time point, protein-containing aliquots were replaced by fresh SBF. The type I collagen level was measured using a MicroVue sandwich ELISA kit (Quidel California, USA), which detects the carboxy-terminal propeptide of type I collagen (CICP). Whereas, the laminin concentration was determined using a QuantiMatrix ELISA kit (Millipore, Cork, Ireland), which employs the principal of competitive antibody inhibition and chromogenic detection. The light absorbance of the colored end solution from above coatings was measured at 405 and 450 nm, respectively. A standard colorimetric curve was used to calculate the ECM proteins concentration. The results present an average of three analyses. The replacement of aliquots with fresh SBF was taken into account for calculation.

### Stem Cell Cultures

This study used the StemPro® human bone marrow-derived MSCs and StemPro® human fetal brain-derived NSCs (Thermo Scientific, Surrey, UK) as cellular models. Unique to these cells is the low-oxygen manufacturing process in which they are isolated and expanded, resulting in higher yields of potent stem cells (Vertelov et al., 2013). They were cultured at 37°C in a humidified 5% CO_2_ atmosphere in a commercial complete cell culture medium. For the MSCs culture, the MesenPRO RS™ complete medium with a Dulbecco's modified Eagle's medium (DMEM)-based and reduced-serum formulation was used. In order to avoid reduced multipotency of MSCs, cells were passaged when they reach 70% confluency, cell viability was at least 90%, and the growth rate was in mid-logarithmic phase. Cells were harvested according to the company's protocol. The MSCs were differentiated into osteogenic lineage in the StemPro® osteogenesis differentiation kit containing ascorbic acid and dexamethasone.

In contrast, the NSCs were maintained in suspension culture for proliferation because plating NSCs on a matrix as an adherent culture would trigger differentiation. The complete NSC culture medium contained 97% DMEM, 2% StemPro® neural supplement, 20 ng/ml basic fibroblast growth factor (bFGF) and 20 ng/ml epidermal growth factor (EGF), 2 mM L-alanyl-L-glutamine, 6 units/ml heparin, and 200 μM ascorbic acid (Sigma-Aldrich). The NSCs were harvested using Accutase® (Sigma-Aldrich) cell dissociation reagent to separate the neurospheres in the suspension culture system. Spontaneous neural differentiation of NSCs was allowed using the differentiation medium which consisted of complete NSC culture medium without bFGF and EGF. Passages 3–8 of stem cells were used to minimize change in growth pattern by *in vitro* expansion. Cellular experiments on coated and non-coated implants were performed in 6-well tissue culture plates (Sarstedt) with each well-immersed in 3 ml of medium.

### Cell Attachment and Adhesion

Cell attachment was analyzed quantitatively and qualitatively using flow cytometry and confocal laser scanning microscopy (CLSM), respectively. The number of attached cells was quantified using the Cell Lab Quanta SC flow cytometry system (Beckman Coulter Inc., Florida, US), employing propidium iodide (PI) as a fluorescent DNA dye. Stem cells were inoculated onto the implants at a concentration of 8 × 10^5^ cells/ml. This relatively high concentration was used to saturate the bonding capacity of each surface to prevent falsely low results. After 12 h incubation at 37°C, unattached cells were gently rinsed away with Dulbecco's phosphate buffered saline (DPBS). Following enzymatic detachment, cells were carefully collected and counted by flow cytometer.

After 12 h of culture, MSCs were fixed *in situ* with 4% (w/v) paraformaldehyde DPBS solution, gently washed twice with wash buffer containing 0.05% (v/v) Tween-20, permeabilized with 0.1% (v/v) Triton X-100 solution, and blocked with 1% (w/v) bovine serum albumin (BSA). The immunofluorescent staining was completed using the actin cytoskeleton and focal adhesion staining kit (Millipore, Watford, UK). In brief, 1% (v/v) primary anti-Vinculin antibody, 1% (v/v) fluorescein isothiocyanate (FITC)-conjugated secondary antibody, and 1% tetramethylrhodamine isothiocyanate (TRITC)-conjugated phalloidin were used successively. The stained MSCs were observed using an LSM 510 Meta confocal laser scanning microscope (Carl Zeiss Ltd., Cambridge, UK).

Cell adhesion on the implants was measured via cell detachment by Accutase. Prior to detachment, the stem cells were inoculated at a concentration of 1 × 10^6^ cells/ml to saturate the bonding capacity of the substrate. Over-seeded cells were gently rinsed away with warm DPBS. Following 24 h of incubation, the adherent cell number was determined using alamarBlue assay (Invitrogen, Paisley, UK), which is an *in situ*, non-toxic metabolism-based cell counting method. Then 3 ml of Accutase was added to each well and incubated for 2 min at 37°C. After enzymatic detachment, floating cells were discarded, and residual cells were collected and counted. The cell viability was always over 90% after cell adhesion assay. Fraction of adherent cells is defined as the post-detachment cell number divided by the pre-detachment cell number.

### Cell Cycle and Proliferation

Human MSCs and NSCs were inoculated onto the non-coated and coated implants at a density of 2 × 10^5^ cells/ml. This relatively low concentration was to prevent the cells from reaching confluency too soon. As previously mentioned, MSCs and NSCs used in this study were maintained in different culture types: adherent culture vs. adherent/suspension culture, respectively. Therefore, different approaches were taken during medium change and cell quantification in each implant-containing well of the tissue culture plate. For the MSCs groups, only the adherent cells on the implants were collected. Whereas, for the NSCs groups, both the adherent cells and those suspending in the medium were harvested. The spent cell culture medium was replaced every 2 days. At each time point, cells were enzymatically detached from the implants, carefully collected, and counted using flow cytometry.

In order to simplify the comparison of cell cycle distribution, only the negative control and test group were used. After the initial cell seeding, a sufficient volume of medium was added on day 1 so that nutrient deprivation would not occur even after 5 days of continuous culture (Tan et al., 2012b). In short, the collected stem cell pellet was mixed with hypotonic DNA staining buffer (0.1% sodium citrate, 0.3% v/v Triton-x100, 0.01% PI, and 0.002% ribonuclease A in dI H_2_O; all from Sigma-Aldrich), and analyzed using a C6 flow cytometer (Accuri, Cambridge, UK) which was equipped with FlowJo 9 software (TreeStar Inc., Oregon, USA).

### Stem Cell Differentiation

The osteogenic differentiation from MSCs and neurogenic differentiation from NSCs were measured after culturing in corresponding differentiation media for 14 days and 10 days, respectively. The osteogenic capacity was reflected by calcium deposition in the secreted mineral matrix. On day 14, the MSCs were decalcified with 0.6 M HCl for 24 h. The calcium content of the HCl supernatant was quantified colorimetrically, using the *o*-cresolphthalein complexone method (Sigma-Aldrich) (Mori et al., 1998). Following decalcification, the MSCs were washed three times with PBS and solubilized with 0.1 M NaOH/0.1% sodium dodecyl sulfate (SDS). The intracellular protein content was estimated using a bicinchoninic acid (BCA) protein assay kit (Sigma-Aldrich), and the amount of calcium was normalized to the total protein content.

On the other hand, the neurogenic competence was demonstrated by neurite formation. Since the PS substrate used was naturally transparent, the NSCs-grown implants were examined with a DM 500 light inverted phase contrast microscope (Leica, Milton Keynes, UK). On day 10, eight random locations (x100 magnification ratio) were chosen from each sample. The number of differentiated neurons and the total cell number in each visual field were counted. The cells with one or more neurites were considered differentiated neurons (Xiong et al., 2014). The differentiation ratio was quantified as the percentage of neurons.

### Pathway Expression Analysis

On day 12 of osteogenic and neurogenic differentiation, the target mRNA strands were reverse transcribed into their DNA complements (cDNA) and amplified using a reverse transcription polymerase chain reaction (RT-PCR). The RNA was extracted and purified using the RNeasy Plus Mini kit (QIAGEN, West Sussex, UK), which was followed by a quality check of total RNA using an ND-1000 NanoDrop spectrophotometer (Thermo Scientific) and an Agilent 2100 Bioanalyzer (Agilent Technologies, Cork, Ireland). Then cDNA was synthesized by using the RT^2^ PCR array First Strand Kit (SABiosciences, Frederick, USA) to ensure compatibility with the subsequent PCR array analysis.

The human osteogenesis and neurogenesis RT^2^ Profiler PCR Array (SABiosciences) were used to profile the expression of a focused panel of genes related to the pathways of osteogenesis/MSC differentiation and neurogenesis/NSC differentiation, respectively. This system used a 7900HT Fast Real-Time PCR (Q-PCR) machine (Applied Biosystems, Cheshire, UK) with 96-well plates. Each plate contained primers for 84 target genes and 5 housekeeping genes, 1 control to rule out human genomic DNA contamination, triplicates of the reverse transcription control to confirm RNA quality, and triplicates of positive PCR control to quality-control the general PCR performance (Tan et al., 2012b). The RT^2^ Q-PCR master mix containing SYBR Green/ROX was mixed with cDNA and aliquoted into 96 wells. PCR cycling was performed with the following thermal profile: 1 cycle of 10 min at 95°C for enzyme activation, 40 cycles of 15 s at 95°C for denaturation, and 1 min at 60°C for annealing and extension. The RT^2^ PCR Array Data analysis Web Portal (SABiosciences) was used to import cycle threshold (*C*_T_) values to enable data calculation. The fold change and fold up/down regulation (test group vs. negative control) was calculated using the 2^−ΔΔ*CT*^ method (Livak and Schmittgen, 2001). Statistical comparison of the mean *C*_T_ values originated from the triplicates of each implant specimen, expressed as *P*-value, was calculated using the Student's *t*-test. In this study, gene expression was considered significantly altered only when two conditions were simultaneously met: the fold regulation >2 or <-2, and the *P*-value <0.05 (Tan et al., 2012b).

The data of our pathway-focused gene analysis was verified at the protein level. A total of 8 genes from the above PCR array results were picked to represent up- and down-regulated genes during the osteogenesis and neurogenesis differentiation. The Quantikine® colorimetric sandwich ELISA kits (R&D Systems, Abingdon, UK) quantified the translated proteins from these genes. The analytes and standards were immobilized and sandwiched by capture antibodies and detection antibodies, respectively. Streptavidin-HRP was then used to bond the detection antibodies, and substrate solution was added to develop colors. The reaction was terminated using the acidic stop solution, and the optical density of each well was determined immediately at 450 nm (Ketelaar et al., 2016).

### Statistical Analysis

Student's *t*-test was conducted for one-to-one comparison unless stated otherwise. A statistical difference was claimed when the *P* value was <0.05. In order to signify the statistical differences in tables and figures, a single asterisk was used when comparing a test group or positive control with a negative control, and a double asterisk, when comparing a test group with a positive control). All statistical calculations were performed using SPSS Statistics 25 (IBM®, Chicago, IL).

## Results and Discussion

### Material Characterization of the Coated and Non-coated Implants

This study assessed the material characteristics of surgical implants before and after coating as to four aspects: chemical properties, physical properties, morphological appearance, and biological stability. XPS revealed distinct shifting in the surface chemical composition when comparing Ti and PS implants (Table 1). The native Ti-6Al-4V substrate exhibited an abundant TiO_2_ layer along with possibly either a hydrocarbon layer on top or carbon contamination from the passivation process (Sittig et al., 1999). The minor presence of aluminum on the surface was most likely due to residuals from the micro-blasting process, which used Al_2_O_3_ to roughen the surface (Milošev et al., 2000). Vanadium is not usually detected in spontaneously formed surface oxides (Ask et al., 1990). After coating the Ti implant with collagen, a substantial increase in carbon (from 33.5 to 60.4%) and nitrogen (from 1.2 to 15.9%) was noticed, indicating a robust layer of amino acid-based protein molecules. The dramatic reduction of titanium (from 18.1 to 1.9%) suggests a reasonable coverage of the collagen coating over the implant surface. These elemental changes are very similar to collagen coatings realized by other methods (Morra et al., 2006; Scarano et al., 2019). By comparison, even though polystyrene's chemical formula is (C_8_H_8_)n, the native PS surface is seemingly composed of carbon only (Table 1). This is because hydrogen cannot be directly detected using the XPS technique. After coating the PS implant with laminin, the strong appearance of nitrogen (6.7%) was accompanied by a considerable drop in carbon (from 98.7 to 80.4%). Laminin is a macromolecule with the skeleton of the molecular chain mainly consisting of C and N; and previous work has shown that the C/N ratio of laminin layer is around 12 (He et al., 2013). This ratio is identical to our finding, denoting the successful deposition of the laminin layer.

**Table 1:** Quantitative XPS analysis of Ti substrate with and without collagen coating, and PS substrate with and without laminin coating (Data is shown as atomic % elemental composition).

**Atomic %**	**Ti implants**		**PS implants**	
	**Non-coated**	**Coated**	**Non-coated**	**Coated**
O	43.9	21.7	1.0	12.9
C	33.5	60.4	98.7	80.4
N	1.2	15.9	0.3	6.7
Ti	18.1	1.9		
Al	3.3	0.1		

*Hydrogen atoms are neglected*.

**Figure 3:**
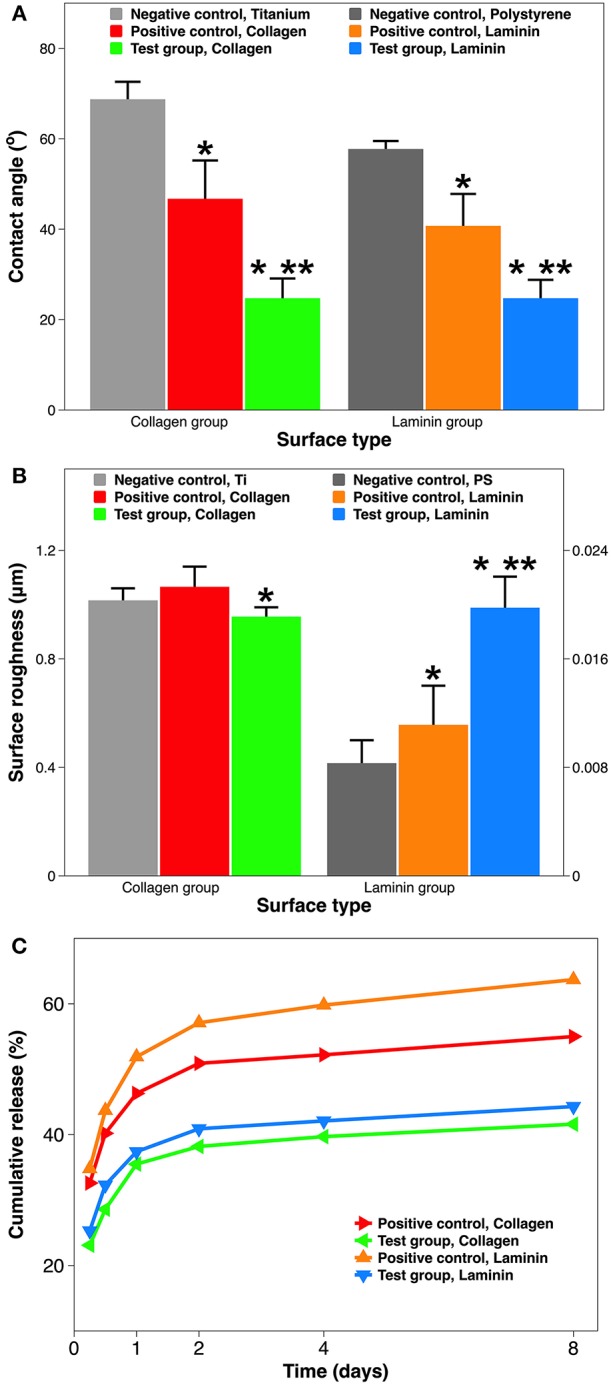
Material characterization of implant surface with and without ECM protein coatings. **(A)** Surface hydrophilicity measured by goniometry (expressed as water contact angle in SBF). **(B)** Surface roughness measured by profilometry (expressed as *Ra* in μm). **(C)** Dissolution of ECM coatings over 8 days, quantified using ELISA (expressed as % cumulative release). A single asterisk denotes statistical significance when comparing a test group or positive control with a negative control, and a double asterisk when comparing a test group with a positive control.

Surface wettability is another important chemical property of implant material. In order to predict the implant wettability under physiological conditions, SBF was used instead of DI H_2_O during contact angle goniometry (Tan et al., 2012b). Both Ti and PS surfaces are considered hydrophilic, as they have water contact angles are <90° (Zeiger et al., 2013; Strnad et al., 2016). Coating these implants with ECM proteins seems to enhance their hydrophilicity, with a greater effect seen in the plasma-facilitated coatings (from 65° to 25°) (Figure 3A). Although coatings by adsorption reduce the contact angle to ~45°, this results in a much larger standard deviation, indicating a less homogenous surface. It is well-known that hydrophilic surfaces tend to provide superior stem cell behavior than hydrophobic surfaces (Ahn et al., 2014; Hao et al., 2014; Yang et al., 2017). In addition, among surfaces with various degrees of hydrophilicity, moderately hydrophilic ones (20°−40° water contact angle) might render optimum cell attachment, spreading, and cytoskeletal organization (Webb et al., 1998). Thus, using atmospheric plasma to apply collagen and laminin coatings have the potential to create more favorable surfaces for MSCs and NSCs.

Surface roughness is a key physical property of implant material. Moreover, roughness and hydrophilicity are two independent but synergistic material properties for implant-related biocompatibility (Ponsonnet et al., 2003). Using optical profilometry, surface roughness is reflected by *Ra* which is the average distance from an arithmetic center line. The Ti and PS implants are considered relatively rough and smooth, respectively, as their surface roughness are at the micro-scale and nano-scale (1.21 μm vs. 8.5 nm). However, the effect of collagen or laminin coating on surface roughness is curiously opposite (Figure 3B). For the Ti implant group, adding a collagen coating by atmospheric plasma marginally reduces the surface roughness (from 1.02 to 0.96 μm). On the contrary, for the PS group, coating using the plasma with laminin significantly increases the surface roughness (from 8.4 to 19.9 nm). This unexpected change is best explained by the nanometer dimension of ECM proteins (Sharma et al., 2019). Depending on the roughness scale, nm proteins could “shallow” rougher surface and “deepen” smoother surface, respectively. Exciting recent studies demonstrate that, either an average roughness of 0.93 μm alone (Faia-Torres et al., 2015), or collagen itself (Lan and Wang, 2003), can serve as compelling alternatives to osteogenic supplements in stimulating osteogenic differentiation of MSCs.

**Figure 4:**
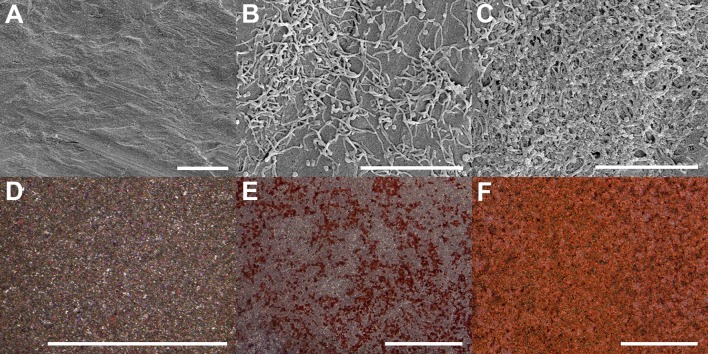
Surface morphology of Ti implants with and without collagen coatings visualized using SEM **(A–C)** and Sircol staining **(D–F)**. **(A,D)** Ti substrate only. **(B,E)** Coating by adsorption. **(C,F)** Coating by atmospheric plasma. Scale bar = 5 μm **(A–C)** & 1 mm **(D–F)**, respectively.

The surface morphology of non-coated and coated implants was observed using SEM and functional staining (Figure 4). The Ti substrate exhibited a typical topography after grit-polishing and micro-blasting (Figure 4A). Its surface was carved with unidirectional linear grooves which are from polishing with sand paper, and decorated with random troughs and peaks which are from Al_2_O_3_ blasting. In addition, little residual micro-grit could be found on the surface. Collagen coatings by adsorption and plasma shows completely different morpholoies. The plasma-facilitated collagen coating is dense, compact, multilayered, and homogenous (Figure 4C). On the contrary, the adsorbed collagen coating is thin, loose, disorganized, and heterogenous (Figure 4B). Zooming out from the μm-scale SEM analysis to the mm-scale Sircol collagen staining, again they exhibit obvious differences. The non-coated Ti substrate does not seem to be stained, and displays a typical grainy and randomly reflective morphology (Figure 4D). The adsorbed collagen coating has rather a patchy and incomplete collagen clusters, and is only partially stained (Figure 4E). The plasma-facilitated collagen coating, on the other hand, demonstrates the typical tint of Sirius red, and is completely colored (Figure 4F). In addition, atmospheric plasma deposited 5.3 times more collagen than the adsorption method. These results support coating by atmospheric plasma as the more robust method, and agree with the findings on collagen coatings created using other advanced techniques (Truong et al., 2012; Kim and Kim, 2016).

Coating dissolution in SBF is a reliable predictor for its durability in physiological conditions. Figure 3C demonstrates the cumulative amount of collagen and laminin released in SBF over an 8-day period. All samples shared a similar release kinetics: a burst release in the first day, followed by a slower release over the next week. The comparison among coatings is 2-fold: collagen coating vs. laminin coating, and coating by adsorption vs. coating by atmospheric plasma. Firstly, a collagen coating has a slower release than laminin coating, especially when they are deposited by simple immersion (55.0 vs. 63.7% at day 8). This most likely occurs due to the coating coverage on the substrate. Coatings applied by simple adsorption are incomplete, resulting in much greater exposure of the underlying Ti or PS substrate (Figure 4). Meanwhile, commercial Ti has a better protein adsorption than tissue culture PS (Derhami et al., 2001). Thus, the difference in substrate implants translated into the difference in coating dissolution. Secondly, coating by atmospheric plasma has a slower dissolution than coating by adsorption (41.6 vs. 55.0% at day 8). We suspect the key to this advantage comes from the plasma activation step during the coating process. Atmospheric plasma is a partially ionized low-pressure gas comprising of ions, electrons, ultraviolet photons, and reactive neutral species including radicals and excited atoms and molecules (Breathnach et al., 2018). Plasma activation bombards the implant surface with these ions and radicals inserting reactive functionality. The advantage of this surface biotechnology includes increased surface wettability without any structural damage (Tan et al., 2012b) and improved protein adsorption on the implant surface (Stallard et al., 2012). Thus, the resultant surface is suitable for crosslinking or subsequent deposition of organic and bioactive agents (Hauser et al., 2010). A recent study of collagen coating showed that introducing more reactive -OH groups on the surface can increase the amount of covalently bonded collagen (Hum and Boccaccini, 2018). It should be noticed that the initial burst release of the ECM coatings is still significant (35.5% of collagen and 37.4% of laminin). This is because the plasma activation only alters ECM proteins close to the surface; those not covalently bonded to the surface still diffuse relatively fast.

Collectively, ECM protein coatings facilitated by atmospheric plasma are more chemically hydrophilic, biologically rough, morphologically robust, and physiologically durable, than their counterparts deposited by simple adsorption. The interaction between these coatings and stem cells will be probed at cellular, protein, and genetic levels in the subsequent experiments.

### Stem Cell Attachment and Adhesion

**Figure 5:**
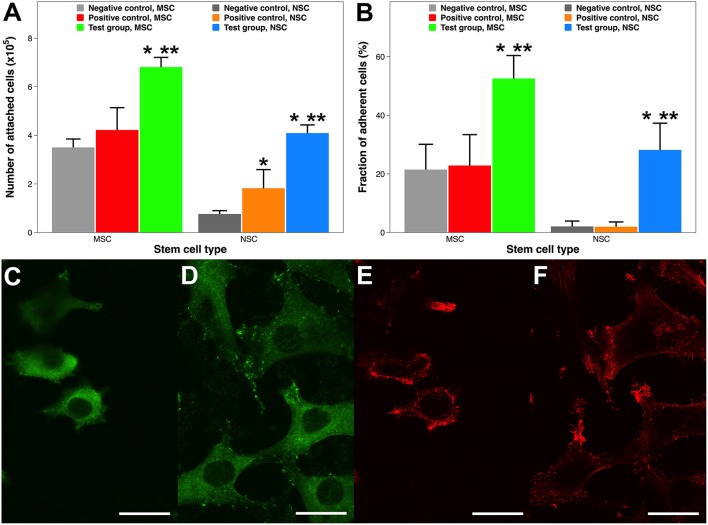
Stem cell attachment and adhesion on surgical implants with and without ECM protein coatings. **(A)** Quantitative evaluation of MSC and NSC attachment 12 h after inoculation. **(B)** Adherence fraction of stem cells after enzymatic detachment. **(C–F)** CLSM images of MSCs morphology. **(C)** Vinculin focal adhesion on Ti substrate. **(D)** Vinculin focal adhesion on collagen coating by atmospheric plasma. **(E)** Actin cytoskeleton on Ti substrate. **(F)** Actin cytoskeleton on coating by atmospheric plasma. Scale bar = 15 μm. A single asterisk denotes statistical significance when comparing a test group or positive control with a negative control, and a double asterisk when comparing a test group with a positive control.

Human stem cells were seeded onto the Ti and PS implants, incubated for 12 h, and counted using flow cytometry. Unlike MSCs, an adherent cell line, NSCs can be grown either as floating 3D neurospheres or attached 2D monolayers on specially coated plates (Conti et al., 2005). Therefore, enhancing cell attachment by ECM protein coatings has a greater importance for MSCs than for NSCs. Compared to non-coated Ti samples, collagen coating by atmospheric plasma nearly doubles the MSC attachment (6.84 vs. 3.53 × 10^5^ cells) (Figure 5A). However, there is no statistical difference between cell attachment on Ti only and coating by adsorption. Collagen is well-known for better promoting attachment and the subsequent proliferation of MSCs (Somaiah et al., 2015). But it only recently became clear that surface matrix has a much more profound effect than induction medium on MSC cellular events (He et al., 2017). In other words, an *in-situ* collagen coating is more potent than diffused collagen during MSC attachment. Similarly, coating a PS surface with laminin by either technique also dramatically enhances NSC attachment, as NSC attachment on non-coated tissue culture PS is minimal (0.79 × 10^5^ cells). In addition, laminin coating by atmospheric plasma attached more than twice as many cells than that by adsorption (4.12 vs. 1.85 × 10^5^ cells). It is a common practice to coat tissue culture containers with attachment proteins such as laminin and poly-L-lysine in laboratory and industrial settings to improve stem cell attachment and proliferation (Lam and Longaker, 2012). Thus, our findings on plasma-facilitated ECM coatings possess huge potential for regenerative medicine and related disciplines.

Stem cell attachment was also qualitatively examined using CLSM 12 h after the initial inoculation, when cells on all surfaces were similarly settled (Tan et al., 2011). Cell attachment is reflected by the distribution of intracellular actin filaments and extracellular vinculin focal adhesion. MSCs attached onto the Ti substrate and collagen coating by atmospheric plasma exhibited substantially different morphologies (Figures 5C–F). Firstly, fewer cells are attached on the Ti surface, which is consistent with the findings in Figure 5A. Secondly, cells attached on the collagen coating had more well-defined, robust, and dotted vinculin-containing focal adhesion plaques (Humphries et al., 2007), which are patterned along the entire edge of the cells helping them to anchor to the implant surface (Figure 5D). Thirdly, cells attached onto the collagen coating are more relaxed and are dispersed with a sturdy actin network consisting of cortical and radial fibers (Figure 5F). Thus, cell attachment is morphologically better on collagen coatings.

Cell adhesion is a relatively static state compared to cell attachment, which is a dynamic process. Various methods have been established to test cell adhesion on surgical implants (Tan et al., 2012a,b; Fong et al., 2017); this study uses an enzymatic one. Around half of the MSCs still remained attached on the collagen coating facilitated by plasma, whereas nearly 80% of cells were dissociated on the Ti and collagen coating by adsorption (Figure 5B). Comparably, almost all the NSCs are detached from the PS surface and the laminin coating by adsorption, while roughly a third of cells remain adhered to the laminin coating by atmospheric plasma. Therefore, cells adhered much more firmly on ECM protein coatings created by atmospheric plasma.

Collectively, ECM protein coatings facilitated by atmospheric plasma supported superior cell attachment and adhesion. This enhancement might be multifactorial: such a coating not only features better chemico-physical material properties, such as hydrophilicity (Paital and Dahotre, 2009) and roughness (Ponsonnet et al., 2003), but also furnishes biologically adhesive functionality (Hagbard et al., 2018). It should be noticed that attachment or adhesion not only anchors cells in place but also plays a critical role in stem cell survival and phenotypic maintenance (Flanagan et al., 2006).

### Stem Cell Proliferation and Cell Cycle Distribution

**Figure 6:**
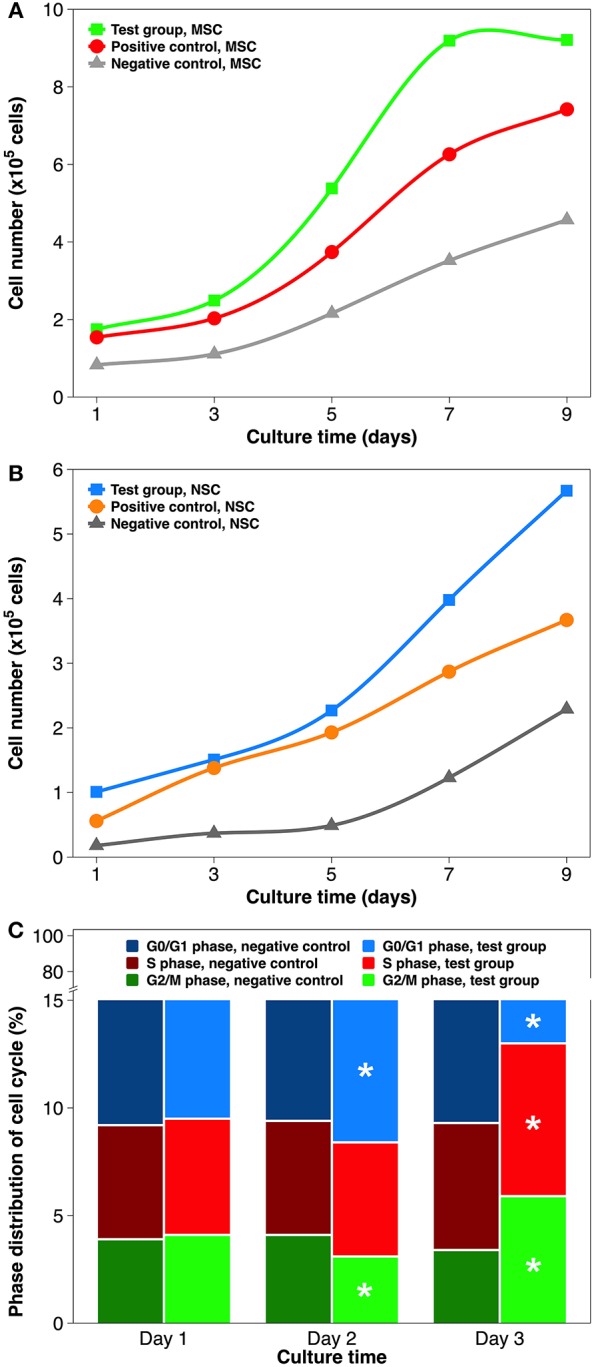
Stem cell proliferation and cell cycle distribution on non-coated and coated surgical implants in non-differentiating culture. **(A)** MSCs growth curve over 9 days of culture. **(B)** NSCs growth curve over 9 days of culture. **(C)** Cell cycle distribution of MSCs grown on the Ti implant and collagen coating by atmospheric plasma, respectively. Statistical annotation is per section Statistical analysis.

Cell growth curves of both stem cell types (Figures 6A,B) exhibit three distinct kinetic patterns. At almost every time point within a 9-day culture, the quantity of grown cells on each surface type was as follows: test group (ECM coating by atmospheric plasma) > positive control (coating by simple adsorption) > negative control (substrate only). In detail, after the initial lag phase, cells on plasma-facilitated ECM coatings entered the exponential growth phase sooner. In addition, cell growth appeared to decelerate on plasma-facilitated collagen coating from day 7, which helpfully signals the start of the stationary phase. It is worth mentioning that the MSCs tended to propagate more quickly than NSCs, which is consistent with the data from the stem cell provider. Based on the values in the exponential phase, cells in the test group coatings had a higher specific growth rate and a lower generation time/doubling time when compared to those on positive control coatings and negative control substrates. In other words, coatings with same ingredient but created by different methods (e.g., atmospheric plasma vs. traditional adsorption) can influence cellular activity to a different extent (Hauser et al., 2010). In any case, reaching cell confluency faster is vital for engineering stem cell-grown implants *in vitro* because MSCs could age sooner than expected, and it is much better to consider them for cell and gene therapy early on (Bonab et al., 2006; Paccola Mesquita et al., 2019).

In order to establish whether the higher cell numbers during cell proliferation followed indirectly from improved cell attachment or was caused directly by elevated growth rate, cell cycle distribution was analyzed by flow cytometry (Figure 6C). The cell cycle comprises of five phases: G_0_ phase (quiescence), G_1_ phase (cell growth), S phase (DNA synthesis), G_2_ phase (continued cell growth), and M phase (mitosis & cytokinesis). The cell cycles are tightly controlled: activation of each phase depends on the progression and completion of the previous one (Sclafani and Holzen, 2007). Therefore, the results of cell cycle phase distribution are best analyzed chronologically. To simplify the comparison, collagen coating by atmospheric plasma and substrate only were chosen. On day 1, the percentages of G_0_/G_1_, S, and G_2_/M phases did not differ significantly. On day 2, slightly more cells were found in the G_0_/G_1_ phase and fewer in the G_2_/M phase, while proving virtually same in the S phase, when comparing test group and negative control. On day 3, all 3 cell cycle phases had statistically different distributions: S phase (7.1 vs. 5.9%), G_2_/M phase (5.9 vs. 3.4%), and G_0_/G_1_ phase (87.0 vs. 90.7%). Overall, using atmospheric plasma to deposit collagen coating on the Ti substrate stimulated MSCs to transit more rapidly from G_1_ to S phase. Cell cycle machinery plays a profound role in the establishment or maintenance of the stem cell state (Morgan, 2007), and is possibly mediated by the timely phosphorylation of focal adhesion kinase (Tan et al., 2012b) and the precocious activity of cyclin-dependent protein kinase (White and Dalton, 2005). The results of NSC cell cycle distribution induced by laminin coating were similar to aforementioned MSCs/collagen group, except that their phase change was slightly delayed due to the slower proliferation rate.

### Osteogenic and Neurogenic Differentiation From Stem Cells

**Figure 7:**
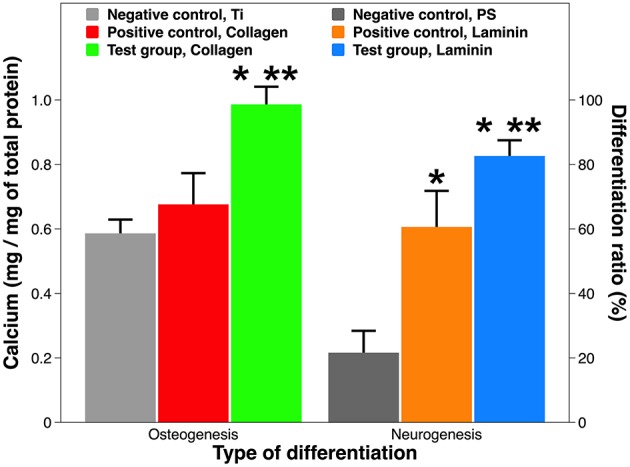
Osteogenic and neurogenic differentiation from stem cells. Osteogenesis is expressed as calcium production by MSCs on Ti implants with and without collagen coatings; neurogenesis is expressed as neuron ratio among NSCs on PS implants with and without laminin coatings. A single asterisk denotes statistical significance when comparing a test group or positive control with a negative control, and a double asterisk when comparing a test group with a positive control.

One defining feature of stem cells is their unique ability to develop into specialized cell types in the body. MSCs are multipotent stromal cells that can differentiate into osteoblasts, chondrocytes, myocytes, and adipocytes (Ullah et al., 2015), whereas NSCs primarily differentiate into neurons, astrocytes, and oligodendrocytes (Bergström and Forsberg-Nilsson, 2012). The target differentiation pathways studied in this work were osteogenesis and neurogenesis (Figure 7). The osteogenic differentiation from MSCs on the Ti substrate and on collagen coatings was reflected by calcium mineralization normalized to the total protein content. No statistical difference was found between the Ti substrate and the collagen coating by adsorption. But the calcium deposition on plasma-facilitated collagen coating nearly doubled that on the metallic substrate (99.7 vs. 58.8%). Comparable results were found on laminin coatings. The non-coated PS surface is a poor candidate to support neurogenic differentiation as just over 20% of NSCs have developed into neurons. Laminin coatings by either deposition technique overcame this disadvantage, increasing the neurogenic ratio up to 83.1%, even though laminin coating by atmospheric plasma still proved superior to its counterpart, simple adsorption.

Our results on tissue-specific differentiation induced by ECM proteins are consistent with peer work. Firstly, MSCs adhere to ECM proteins with varying affinity, resulting in different degrees of osteogenic differentiation: collagen I > vitronectin > laminin (Salasznyk et al., 2004). Interestingly, ECM contact alone may suffice to induce differentiation without osteogenic drugs. Secondly, combining laminin coatings with various surface geometry, topography, and micropatterns might further increase neuronal differentiation (Liu et al., 2006; Christopherson et al., 2009; Xie et al., 2014). Last but not least, mixing various ECM proteins (e.g., Matrigel® and Cultrex®) and co-culturing of MSCs/NSCs might provide synergistic solutions for implant-based therapies (Gattazzo et al., 2014; Yang et al., 2018).

### Pathway-Specific Gene and Protein Analysis

**Figure 8:**
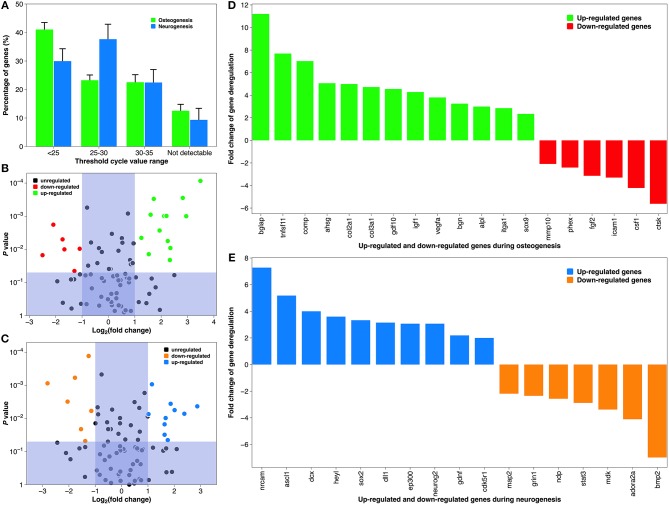
Gene expression during osteogenic differentiation of MSCs and neurogenic differentiation of NSCs. **(A)** The average *C*_T_ distribution of 84 genes studied in the atmospheric plasma-facilitated ECM coating samples. *C*_T_ <25 indicates genes are expressing with high transcript copy number, and *C*_T_ >35 suggests gene expression falls below the detection threshold of the equipment. **(B)** “Volcano plot” of the fold change and statistical significance of the expressed genes during osteogenesis. **(C)** “Volcano plot” of the fold change and statistical significance of the expressed genes during neurogenesis. **(D)** Histogram demonstrating significantly deregulated genes during osteogenesis. **(E)** Histogram demonstrating significantly deregulated genes during neurogenesis. Tick labels in X-axis are composed of gene symbols.

Although the depiction of tissue-specific differentiation here is straightforward, the underlying molecular mechanism can be very complicated (Tan et al., 2012b). Therefore, we conducted pathway-specific gene analysis using a PCR array, which combines the multi-gene profiling capabilities of a microarray with the performance of Q-PCR. Both MSCs on a collagen coating and NSCs on a laminin coating showed constitutive expression of majority of the 84 genes associated with human osteogenesis and neurogenesis, respectively (Figure 8A). But PCR efficiency differed between the two groups, as their predominant *C*_T_ value was distributed differently (<25 vs. 25–30). This was most likely due to differences in the cell lines (Brendel et al., 2005). Comparing the *C*_T_ value of the test group and the positive control, 19 genes during osteogenesis and 17 genes during neurogenesis were deregulated, respectively. The organization of these statistically significantly deregulated genes (fold change >2 or <-2, and *P*-value <0.05), along with the organization of biologically significantly deregulated genes (fold change between −2 and 2 & *P*-value <0.05, and fold change >2 or <-2 & *P*-value >0.05) are best appreciated in the “volcano” charts (Figures 8B,C). A distinct gene expression pattern can be discovered, where the “volcano” axis is shifted to the right during osteogenesis (more genes up-regulated than down-regulated) and centralized during neurogenesis (a balanced number of up- and down-regulated genes). A breakdown of these deregulated genes is illustrated in Figures 8D,E. The fold changes of gene deregulation range from 11.24 (*bglap*) to −5.66 (*ctsk*) during osteogenesis and from 7.31 (*nrcam*) to −7.01 (*bmp2*) during neurogenesis. These deregulated genes are categorized into various functional groups covering all major aspects of osteogenic and neurogenic pathways (Tables 2, 3). We believe that the cause of gene deregulation induced by atmospheric plasma-facilitated ECM coatings is multifactorial, since implant parameters such as surface wettability (Tan et al., 2012b), surface roughness (Brett et al., 2004), collagen (Shekaran et al., 2015), and laminin (Liu et al., 2016) surface modification all can influence the gene expression of interfacial cells. Thus, it would be very intriguing to use atmospheric plasma as a surface biotechnology to combine the above parameters, hoping to achieve a novel implant-based therapy with gene-altering capabilities.

**Table 2:** Functional grouping of up- and down-regulated genes in the osteogenic pathway of human MSCs grown on collagen coatings (coating by atmospheric plasma vs. coating by adsorption).

**Function group**	**Subdivision of function**	**Gene**
Skeletal development	Cartilage condensation	*col2a1, sox9*
	Ossification	*alpl, bglap, col2a1, csf1, fgf2, sox9, tnfsf11*
	Osteoclast differentiation	*bglap, csf1, tnfsf11*
	Osteoblast differentiation	*bglap, fgf2*
	Other	*alpl*
Bone mineral metabolism	Bone mineralization	*ahsg, bglap, sox9*
	Calcium ion binding & homeostasis	*bglap, comp, fgf2*
Extracellular matrix (ECM) molecules	Collagens	*col2a1, col3a1*
	ECM protease inhibitors	*ahsg*
	ECM Proteases	*ctsk, mmp10, phex*
	Other	*alpl, bgn*
Cell adhesion molecules	Cell-cell adhesion	*col2a1, icam1, sox9, tnfsf11*
	Cell-ECM adhesion	*csf1, itga1*
	Other	*bglap*,
Growth factors		*fgf2, gdf10, igf1, vegfa*
Transcription factors		*sox9*

*Full list of genes in the human osteogenesis RT^2^ profiler PCR array can be found on https://www.qiagen.com/gb/shop/pcr/primer-sets/rt2-profiler-pcr-arrays/?catno=PAHS-026Z#geneglobe*.

**Table 3:** Functional grouping of up- and down-regulated genes in the neurogenic pathway of human NSCs grown on laminin coatings (coating by atmospheric plasma vs. coating by adsorption).

**Function group**	**Subdivision of function**	**Gene**
Neuronal migration		*ascl1, dcx, cdk5r1, neurog2, nrcam*
Cell differentiation	Neuronal differentiation	*ascl1, bmp2, cdk5r1, heyl, nrcam, sox2, neurog2*
	Neuronal cell fate determination	*ascl1, sox2*
	Other	*mdk*
Synaptic functions	Regulation of synaptic plasticity	*grin1*
	Synaptic transmission	*grin1*
	Synaptogenesis	*nrcam*
	Axonogenesis	*dcx, map2, nrcam*
Growth factors & cytokines	Growth factors	*gdnf, mdk, ndp*
	Cytokines	*bmp2, mdk*
Apoptosis		*ep300, gdnf*
Cell adhesion molecules		*dll1, nrcam*
Cell cycle		*ep300, mdk*
Signal transduction	Notch signaling	*ascl1, dll1, heyl*
	WNT signaling	*ndp*
	TGFβ signaling	*bmp2*
	G-protein coupled receptor signaling	*adora2a*
Transcription factors & cofactors		*ascl1, ep300, heyl, neurog2, sox2, stat3*

*Full list of genes in the human neurogenesis RT^2^ profiler PCR array can be found on https://www.qiagen.com/gb/shop/pcr/primer-sets/rt2-profiler-pcr-arrays/?catno=PAHS-404Z#geneglobe*.

**Figure 9:**
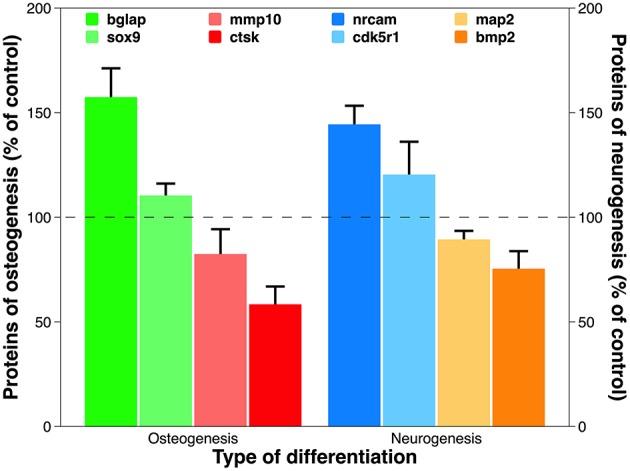
Expression of proteins translated from representative deregulated genes discovered in PCR array analysis for osteogenesis and neurogenesis. Using the optical absorbance at 450 nm during ELISA, the data are presented as percentage of control. Statistical annotation is omitted because the difference between test group and control is statistically significant for each protein tested.

The above transcriptomic data were verified at the translational level. All eight of the representative genes chosen from the above gene assay of osteogenesis and neurogenesis were appropriately translated (Figure 9). Osteocalcin (aka bone gamma-carboxyglutamate protein), encoded by the up-regulated gene *bglap*, is among the most abundant proteins in bone and is produced exclusively by osteoblasts (Zoch et al., 2016). It is a well-known biochemical marker for bone formation, and its level *in vivo* can be affected by the design of surgical implant and post-implantation osteolysis (Qureshi et al., 2002). Cathepsin K, the protein product of the down-regulated gene *ctsk*, is an ECM protease involved in bone remodeling and resorption through osteoclast activation (Costa et al., 2011). Its expression is diminished during bone formation on biomaterials cultured with human MSCs and induced pluripotent stem cells (Jeon et al., 2016). On the other hand, deregulated genes during neurogenesis also reveal transcriptomic authenticity. The neuronal cell adhesion molecule, translated from the up-regulated gene *nrcam*, can induce neurite outgrowth and mediate adhesion among neurons (Weledji and Assob, 2014). Tethering neuronal cell adhesion molecule on biomaterial has become a promising strategy to promote neural adhesion and ultimately, neural tissue regeneration (Rao and Winter, 2009). Bone morphogenetic protein 2, translated from the down-regulated gene *bmp2*, is a potent osteoinductive agent that enhances bone therapy (Poon et al., 2016). Interestingly, it is also heavily involved in the central nervous system where it irreversibly alters NSCs from neurogenesis to undesirable gliogenesis, leading to a failure of neuronal regeneration (Nakashima et al., 2001).

To recapitulate, collagen and laminin coatings created by atmospheric plasma can significantly alter the gene expression of MSCs and NSCs during osteogenesis and neurogenesis, respectively. This improvement over coatings using the traditional technique is reflected within multiple functional pathways among tissue-specific differentiation. The 36 deregulated genes discovered in this study can be used as potential targets to design treatments delivered by surgical implants. One option with great promise for clinical therapies would be biomaterials capable of localized gene delivery that synergistically target multiple cell processes, leading to the regeneration of many tissues (Gower and Shea, 2013).

## Conclusions

Extracellular proteins were coated onto surgical implants using the atmospheric plasma. Coatings formed by this novel technique were compared with those created by the conventional adsorption method. The study compared how both methods affected material characteristics and biological interactions with stem cells. XPS, SEM, and functional staining collectively revealed that atmospheric plasma created complete and homogeneous coatings. The resultant collagen and laminin coatings proved more hydrophilic *in vitro* and potentially more durable *in vivo*. Quantitative and qualitative assessment confirmed the superior mesenchymal and NSC attachment and adhesion on plasma-facilitated coatings. In addition, cell proliferation was independently faster, as verified by the cell cycle distribution analysis. Finally, tissue-specific differentiation from mesenchymal and NSCs was also significantly boosted by our newly designed ECM coatings. The underlying molecular mechanism was probed using pathway-specific PCR array. A total of 36 genes were discovered to be deregulated during osteogenesis and neurogenesis. The overall transcriptomic advantage of these promising coatings was evidenced throughout diverse functional pathways. Altogether, we have demonstrated the versatility of atmospheric plasma as a surface biotechnology, since it produces an implant-specific coating which offers tissue-specific enhancement. Future work could include using atmospheric plasma for implant-based drug delivery in regenerative medicine.

## Data Availability Statement

All datasets generated for this study are included in the manuscript/Supplementary Files.

## Author Contributions

FT designed, performed the experiements, analyzed the data, and wrote the manuscript. MA-R corrected the manuscript.

### Conflict of Interest

The authors declare that the research was conducted in the absence of any commercial or financial relationships that could be construed as a potential conflict of interest.
